# Students' Digital Competence and Perceived Learning: The mediating role of Learner Agility

**DOI:** 10.12688/f1000research.124884.1

**Published:** 2022-09-12

**Authors:** Vidya Patwardhan, Jyothi Mallya, Rahul Shedbalkar, Sandeep Srivastava, Kartikeya Bolar

**Affiliations:** 1 Welcomgroup Graduate School of Hotel Administration, Manipal Academy of Higher Education, Manipal, Udupi, Karnataka, 576104, India; 2 T A Pai Management Institute, Manipal Academy of Higher Education, Manipal, Udupi, Karnataka, 576104, India

**Keywords:** Digital competence, learners' agility, perceived learning

## Abstract

**Background:** The ravages of COVID-19 escalated the penetration of online education and usage of digital technologies. While educational institutions across the globe adopted different forms of computer-mediated communication, the institutes in India have gradually attuned to the new normal, notwithstanding the initial glitches of adopting new technology and shifting to blended. It became increasingly significant to gain a better understanding of students’ perspectives of newly emerged learning environment. This motivated the researchers to study the digital competencies (DC) and their impact on students’ learning agility (LA) and perceived learning (PL) in professional/technical education.

**Methods:** In this cross-sectional study, a DigiComp 2.1 framework was attempted to investigate the relationship between DC and PL among higher education students in India. The data from 359 graduate and post-graduate students were analyzed using Structural equation modelling and Process Macro 4.0.

**Results:** The findings of this study revealed that DC has a significant positive impact on PL (b = 0.33; p < 0.001), indicating that higher learners’ DC leads to higher learning outcomes. Similarly, DC also had a significant positive impact on LA (b = 0.59; p < 0.001), suggesting that the higher DC of learners leads to higher learning agility. Further, a positive significant relationship was also found between LA and PL (b = 0.21; p < 0.001). This significant positive path reveals that higher learners’ agility leads to higher student learning outcomes.

**Discussion:** Post-COVID, DC, a technology-related skill set is linked to the academic performance of teachers and students. Our findings reveal that DC significantly positively impacts PL and LA. Therefore, we recommend that the higher educational institutes in India consider the inclusion of DC in their curriculum as a fundamental competence for a better learning outcome for learners.

## Introduction

With the unprecedented entry of COVID-19 into our lives, digital technologies are re-evolving and emerging as one of the most potent tools even in the most non-volatile ecosystem of education. Today, education is broken, and we are trying to fix it with technology (technologization) ( Teräs *et al.*, 2020). This profound change toward democratization of education expects high levels of digital competence (DC) from teachers and students. Though it started as a stopgap solution due to the COVID crisis, the technology dependence spearheaded the abrupt shift toward full-fledged online education ( OECD, 2020). This shift necessitates proficiency in a series of DC for learning and performance in digital formal and informal learning environments ( Elstad & Christophersen, 2017; Heidari *et al.*, 2021; Mehrvarz *et al.*, 2021). Critical to the success of the transition to online education is the inevitability of attaining the requisite knowledge, skills, and attitudes to embrace digital technologies constructively ( Coman *et al.*, 2020; OECD, 2020). It is a tectonic shift ( Govindarajan & Srivastava, 2020) featuring hybrid or blended classrooms, collaboration, equity, experimentation, and innovation that may continue to be an effective learning ecosystem ( Miroshnikov, 2021). Numerous online resources facilitated students to access, create, and share digital content for collaborative education. The role of DC has become more critical due to its holistic emphasis on the ethical, safety, and social dimension and the inclusion of diverse knowledge, abilities, and desires of individuals ( Falloon, 2020; Foulger *et al.*, 2017). Parallel direction is apparent within the education domain, where the focus should be on enhancing the learner’s capabilities for better participation in digital society ( Martzoukou *et al.*, 2020).

The development of digitally competent, able, and skilled professionals within the ever-changing technological and online environment expect learners to be agile in their ability to learn, adapt, unlearn, and relearn to keep up with the frequently changing learning environment ( Fulton & McGuinness, 2016; Martzoukou *et al.*, 2020). The digitally literate generation must remember the three vital components of learning agility (LA): 1. Potential to learn, 2. Motivation to learn, and 3. Adaptability to learn ( Amato & Molokhia, 2016). Agile learners are willing to learn continuously and apply the knowledge in new situations ( De Meuse *et al.*, 2010; Kim *et al.*, 2018). In a post-COVID academic environment, it is extremely important to be agile in the adoption of technologies that allow for flexible and personalized learning ( OECD, 2020). Today, governments, institutions, educators, and students have experienced the need for digital literacy and generic digital skills. However, past research shows that undergraduate students need intense training in digital technologies as they do not effectively attempt to integrate them into their educational experiences ( Piotrowski, 2015; Strømsø *et al.*, 2013).

The key terms used to explain digital technologies in digital parlance include information and communication technology (ICT) literacy, Internet skills, Information literacy, media literacy, digital literacy, and DC ( Chetty *et al.*, 2018). Among these, DC, an emerging concept that describes technology-related knowledge and skills, has been acknowledged as a critical competence vital for enduring learning ( Falloon, 2020; Iordache *et al.*, 2017). In the higher education research context, it is defined as “the ability to explore and face new technological situations flexibly, to analyze, select and critically evaluate data and information, to exploit technological potentials to represent and solve problems and build shared and collaborative knowledge, while fostering awareness of one’s responsibilities and respect of reciprocal rights/obligations” ( Scuotto & Morellato, 2013; Spante *et al.*, 2018). Due to the advent of continued online learning, DC has become a buzz term that resonates explosion of digital information, communication, and interaction among people, especially the academic fraternity. According to the European DC framework for citizens (DigComp 2.1), the five key components of DC are; 1. Information and data literacy, 2. Communication and collaboration, 3. Digital content creation, 4. Safety, and 5. Problem-solving ( Ferrari *et al.*, 2013a). Experts opine that the key components of DC are fundamental to supporting an individual’s lifelong learning and employability ( Guitert *et al.*, 2021; Zhao, *et al.*, 2021). Therefore, the student perspectives of cognitive, emotional and social aspects of the learning process in a digital environment require special attention.

In India, Ministry of Human Resource Development (MHRD) launched various digital initiatives to address the challenge of remote learning to build the future of 25 crore students ( MHRD, 2020). It is time to develop systematic approaches to map the DCs of students in higher educational institutions as a coherent learning continuum. Despite its importance, many higher education institutions in India have not yet developed an organized method to map the DCs of students as a priority. Today, the development of digital skills from the point of view of employability is a baseline requirement. Universities have to design resources to support students to develop digital skills. Using the DigComp 2.1 framework, this study tries to report students’ current DC profile and learning agility that might help bridge the digital divide in institutions of higher learning in India. It is presumed that the extent to which students benefit from digital learning depends on students’ competence in utilizing these environments. As propagated by the developers of DigComp, we need a tool to enhance learners’ DC as a pointer for policymakers to formulate guidelines to improve the DC of specific target groups ( Vuorikari *et al.*, 2016).

Alongside, understanding self-perceived DC levels by the students would facilitate learning as students have diverse digital experiences based on their background characteristics. Hence, the LA of students is taken as a mediator to investigate the effect of DC on students’ PL. It is assumed that LA stimulates the student’s motives to enhance digital skills. This quantitative study aims to test the conceptual framework highlighting the positive relationship between DC, LA, and PL using structural equation modelling and mediation analysis. To the authors’ knowledge, this is an under-researched domain and could be an addendum to continue efforts towards creating a digital society by developing novel DC frameworks specific to the needs of Indian higher education students. Throughout this paper, the term ‘DC’ will be used as an umbrella term for various key terms related to digital skills.

## Literature review

### Digital competence and Perceived Learning

DC is a multi-faceted concept ( Sánchez-Caballé *et al.*, 2020) that evolved from diverse backgrounds ( Gallardo-Echenique *et al.*, 2015; Lucas, 2019). The UK higher education context proposed Digital Capabilities Framework having six elements ( Biggins *et al.*, 2017) that can be used to enhance students’ ability to steer self-learning for continuous development. Likewise, the European Commission developed the DC framework (DigComp2.1) to respond to the ever-increasing need to operate effectively in a knowledge-intensive society ( Sillat, Tammets, & Laanpere, 2021). With five dimensions and 21 elementary competencies, this framework was first published for European citizens in 2013 and renewed in 2017. This framework highlights the significance of digital creation, innovation, communication, collaboration, engagement, and digital identity ( Lucas, 2019; Sillat *et al.*, 2021). Later it was adopted within the education sector to create a standard for evaluating the DC of educators and students ( Lucas, 2019). Experts predict that acceleration in edutech growth will sustain, and DC training in higher education ( MHRD, 2020) will profoundly shift the focus towards using digital technologies to enhance students’ learning experiences and facilitate the development of their DC.

Regrettably, in a traditional learning environment, similar instruction styles are followed regardless of the individual learning abilities of students. The digital resources are designed at baseline, ignoring individual learners’ present DC levels ( Martzoukou *et al.*, 2020). As students belong to different demographics, the requirement of levels of support for DC may vary ( Martzoukou, *et al.*, 2020). The diversity in socio-demographic characteristics may widen the digital divide ( Moore *et al.*, 2018). Hence, it cannot be presumed that all students arrive at university with the same levels of DC. Some studies suggest that students develop DC spontaneously in digital learning environments through active engagement and self-motivation ( Heidari *et al.*, 2021; Lucas, 2019; McGuinness & Fulton, 2019). At the same time, few others emphasize the close linkage between well-founded pedagogy, didactics, and DC ( Sung *et al.*, 2016; Tamim *et al.*, 2011). In the digital learning environment, it is argued that meaningful learning occurs when students are active, constructive, intentional, authentic, and cooperative ( Howland *et al.*, 2012). The above standpoints deliberated by researchers with diverse backgrounds invite the inquiry of learning processes from students’ perspectives ( Blau *et al.*, 2020).

Theoretically PL consists of cognitive, emotional, and social aspects that deal with understanding new insights, feelings and experiences during learning and inter-personal interactions through the learning sessions ( Blau *et al.*, 2020; Rockinson-Szapkiw *et al.*, 2016). It primarily relates to two predominant aspects of learning: knowledge acquisition and knowledge transfer ( Barbera *et al.*, 2013) which are projected to be essential to acquire DCs. However, the prediction of DC having a significant relationship with PL has largely remained unexplored. There is no evidence thus far investigating this relationship in the extant literature related to online education. Hence, we propose the following research hypothesis:

H1: There is a significant positive relationship between students’ Digital competence and perceived learning in an online learning environment.

### Digital Competence, Learner Agility, and Perceived Learning

The researchers in the field of digital literacy and competence feel that mere usage of digital tools will not automatically make students digitally competent ( González & Martín, 2017; Sánchez-Caballé *et al.*, 2020). There is a gap between formal (e.g. educational software, technology theory) and informal (e.g. multimedia tools) digital skills and abilities of university students ( Flores & Roig, 2016; Parvathamma & Pattar, 2013; Prieto *et al.*, 2020; Purushothaman, 2011). In the formal setup, students lack experience in e-learning skills and abilities ( Poulová *et al.*, 2011). Research studies have revealed that undergraduate students need extensive training in digital technologies ( Kim *et al.*, 2018). This training is essential when students enter a blended learning environment, primarily pointing to the post-COVID education scenario. To moderate the gap, in institutions of higher learning, both learners and educators need to develop technology-related knowledge, skills, and attitudes through ongoing learning programmes ( Kim *et al.*, 2018). Only agile ("agile" as used in the domain of technology) methodology and development referring to iterative processes and continuous improvement by building a culture of constant growth ( Himmelsbach *et al.*, 2019) seems to be the viable solution. Students must embrace an agile mindset to meet the demands of digital innovations.

LA is an essential factor that integrates digital technologies into student learning and engagement in academic life. The theory of Learning Agility emphasizes that “individuals who have performed well in the past will not necessarily perform well in the future in a new job” ( Connolly, 2001). It is believed to significantly influence learners’ ability to progress to more complex and challenging learning assignments ( Almeida, 2019). Similarly, it can be presumed that students living in an era of transition may find it challenging to adapt to new learning situations with the present DC levels. Therefore, they are anticipated to be flexible and fast learners amid a high level of knowledge uncertainty posed by COVID-19 and evolving digitalization as prerequisites to seize new opportunities. The construct LA is more appropriate for consideration in this research context as its basis is rooted in adult learning and self-regulated learning ( Allen, 2016). Students perceive that agile practices have a great potential to enhance their learning experiences ( Melnik & Maurer, 2002). The definition of perceived learning, i.e. "changes in the learner’s perceptions of skill and knowledge levels before and after the learning experience", as given by Alavi *et al.* (2002), is appropriate in this context to ensure the quality of learning and improvement in the learning experience. Hence, as a predictor of students’ enriched learning experience, we hypothesize that LA mediates the relationship between DC and PL.

H2: There is a significant positive relationship between students’ Digital competence and learning agility in an online learning environment.

H3: There is a significant positive relationship between students’ learning agility and perceived learning in an online learning environment.

H4: The learning agility of students mediate the relationship between students’ Digital competence perceived learning in an online learning environment.

**Figure 1.**
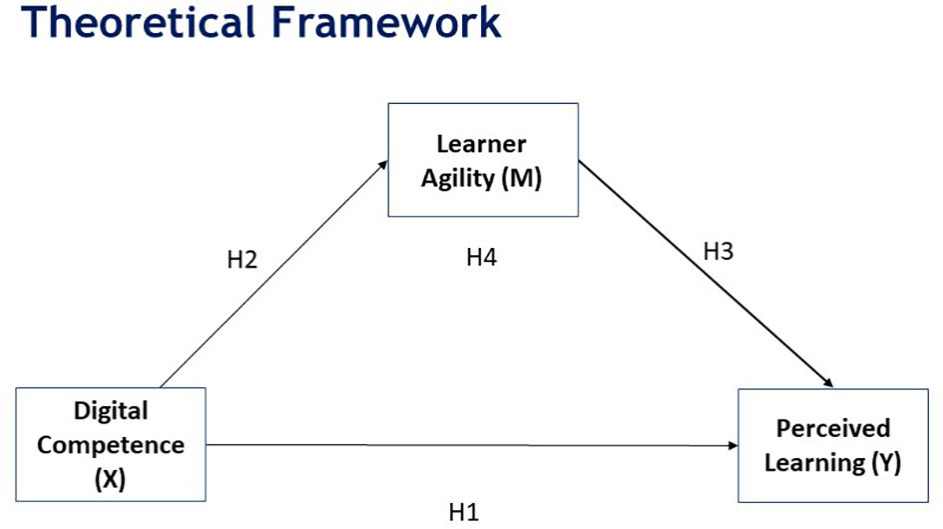
Proposed model.

Based on the above literature, the following model ( Figure 1) is proposed.

## Methods

### Ethics and consent

Ethical approval was obtained from the Institutional Research and Ethical Committee of Welcomgroup Graduate School of Hotel administration (WGSHA), Manipal Academy of Higher Education via Reference No. WGSHA–IRC-2021-02 dated 14-08-2021. The committee waived the written consent since there was no risk involved for the participants, and most participants were above 18 years of age. Parental consent was also waived for a few participants of 17 years because of the no-risk nature of the study, and these underage participants were in the same cohort as the other participants, i.e., university students. Additionally, one of the authors visited the classrooms to explain the objectives and informed the participants that participation in the survey is voluntary. Thus, verbal consent was obtained before distributing the online survey form.

### Data collection and sample profile

Data was collected from 359 full-time students across professional disciplines of a well-known private university in India. This university offers higher education in Medical, Paramedical, Allied Health, Health Science, Pure Science, Technology, Management, Hospitality Management, Commerce, Media, Humanities, Geopolitics, and few other disciplines. The diversity in the background was considered adequate to represent the different proficiency levels in DC among the student community. The questionnaire was developed in Microsoft Forms, and the web link of the online questionnaire was emailed to 1,200 students with an explanation on the constructs as well as study objectives. A week after this, a follow-up email was sent as a reminder to expedite the data collection process. The data was collected in the month of May 2021 and August 2021.

In this cross-sectional research, the respondents were selected based on purposive sampling. The respondents have attended a minimum of 12 months of online classes. In total, 359 valid responses were received yielding a response rate of 30%. The sample included among the respondents, 224 (62.4 %) male and 135 (37.6%) female students. Among the respondents, 315 (87.7%) were undergraduates, and 44 (12.3%) were postgraduates.

### Measurement of constructs

The measuring instrument was developed after an in-depth literature review. The DC survey instrument was borrowed from ( Ferrari *et al.*, 2013b). The 21 items were measured on a 5-point Likert scale where 1 represents “very low”, and 5 represents “very high”. A higher value would indicate a higher level of DC. The LA (five items) was measured based on the scale of Kim *et al.* (2018). Respondents were requested to rate their agreement or disagreement with the statements on a 5-point Likert scale where 1 representing strongly disagree and 5 representing strongly agree. The outcome variable’s PL scale (six items) was adopted from the study by Narayan *et al.* (2021). These variables were operationalized using a 5-point Likert scale ranging from 1 (strongly disagree) and 5 (strongly agree). The respondents’ demographic details such as age, gender, and education were also included in the survey instrument. The full questionnaire can be found in the Extended data ( Mallya & Patwardhan, 2022b).

### Sampling adequacy

The Kaiser-Meyer-Olkin (KMO) test was used to test the sample adequacy. The KMO value is above the recommended value of 0.6 (0.93), and Bartlett’s test of sphericity is significant (χ ^2^ (210) = 4478, p < .001), thus confirming the suitability of data for factor analysis ( Kline, 1994).

### Psychometric properties of the first-order factors

Before assessing the structural model, the first-order factor’s measurement model’s psychometric properties were assessed using the confirmatory factor approach. The model displayed good model fit indices (CFI = 0.95; TLI = 0.94; RMSEA = 0.05; SRMR = 0.05; x2/df = 2.64). The model was further tested for its reliability and convergent validity ( Table 1). Reliability was assessed based on the composite reliability (CR), and convergent validity was assessed based on the average variance extracted (AVE) values. According to Hair *et al.* (2014), the value of CR and AVE should be more than 0.70 and 0.50, respectively. All these values were above the recommended value ( Table 1), suggesting the constructs’ reliability and convergent validity. Further, except for the factor “Communication”, the model achieved discriminant validity ( Table 2). However, this is common due to the high correlation between the manifest indicators ( Koufteros *et al.*, 2009; Marsh & Hocevar, 1985).

**Table 1.** Psychometric properties of the first-order factor measurement scale.

Factors and their indicators	SL	t-value	CR	AVE
Information and data literacy				
INF1	0.756	1	0.836	0.630
INF2	0.811	14.718		
INF3	0.813	14.747		
Communication				
COM1	0.731	13.069	0.874	0.536
COM2	0.729	13.354		
COM3	0.727	12.871		
COM4	0.756	12.904		
COM5	0.739	12.927		
COM6	0.711	1		
Content Creation				
CON1	0.811	1	0.866	0.619
CON2	0.850	14.454		
CON3	0.760	15.057		
CON4	0.720	13.570		
Safety				
SAF1	0.823	1	0.856	0.600
SAF2	0.859	12.324		
SAF3	0.765	12.620		
SAF4	0.632	11.733		
Problem-solving				
PRO1	0.823	1	0.868	0.623
PRO2	0.859	14.608		
PRO3	0.765	17.480		
PRO4	0.632	16.203		

SL – Standardized loadings; CR – Composite reliability; AVE – Average variance extracted.

**Table 2.** Discriminant validity analysis of first-order factor.

	INF	COM	CON	SAF	PRO
INF	0.794				
COM	0.745 ***	0.732			
CON	0.645 ***	0.648 ***	0.787		
SAF	0.440 ***	0.482 ***	0.327 ***	0.775	
PRO	0.649 ***	0.719 ***	0.743 ***	0.595 ***	0.789

*** Significant at 0.001 level.

### Model comparison

After achieving reliability and validity for the first-order factors model, the performance of the second-order factor model of DC was tested. The development of four models using a hierarchical approach was adopted to validate the second-order factor model ( Rindskopf & Rose, 1988). First, the single first-factor model with 21 items of DC was loaded (Model 1). The second model hypothesized that all the five dimensions of DC were separate and unrelated (Model 2). The third model (Model 3) hypothesized that all the five dimensions of DC were distinct but correlated. The fourth model (Model 4) was the second-order factor model of DC.

The hypotheses were tested using confirmatory factor analysis. The results are presented in Table 3. Table 3 shows that Model 1 and Model 2 did not have acceptable model fit indices. Further, Model 3 had marginally better model fit indices than model 4. Though model 3 had better fit indices, model 4, which hypothesizes a second-order factor model, was considered since it also had an acceptable fit.

**Table 3.** Comparison between the four models.

Fit indices values	Model 1	Model2	Model 3	Model 4
X2	1585.849	1251.59	502.65	547.288
CFI	0.680	0.751	0.926	0.917
TLI	0.645	0.730	0.913	0.905
RMSEA	0.144	0.125	0.071	0.0704
x2/df	8.391	6.622	2.808	2.974
AIC	1669.89	1335.58	606.651	641.288
BCC	1832.95	1498.68	808.58	823.80

CFI – Comparative fit index; TLI – Tucker–Lewis index; IFI – Incremental fit index; RMSEA – Root mean square error of approximation; SRMR – Standardized root mean square residual; AIC – Akaike information criterion; BCC – Browne–Cudeck criterion.

## Results

### Measurement model

The overall measurement model was tested using CFA after achieving desired model fit for the second-order factor. The model indices values as per the recommended values (CFI = 0.94; TLI = 0.94; RMSEA = 0.04; SRMR = 0.05; x2/df = 2.37). The second-order factor model of DC was further tested for convergent and discriminant validity. The CR and AVE values were above 0.7 and 0.5, respectively ( Hair *et al.*, 2014) ( Table 4). The discriminant validity of the constructs was tested by comparing the square root of AVE to bivariate correlation values between the constructs ( Table 5). According to ( Fornell & Larcker, 1981) square root of all measuring constructs should be greater than the bivariate correlation values between the constructs. The overall measurement model achieved discriminant validity.

**Table 4.** DC as a second-order factor.

Factors and their indicators	SL	t-value	CR	AVE
Digital competence			0.889	0.619
INF	0.82			
COM	0.859	10.396		
CON	0.858	9.782		
SAF	0.587	7.804		
PRO	0.776	11.161		
Learning agility			0.809	0.515
LEA1	0.655			
LEA3	0.76	11.332		
LEA4	0.693	10.639		
LEA5	0.757	11.301		
Perceived Learning			0.88	0.571
PEA1	0.7			
PEA2	0.732	12.795		
PEA3	0.794	13.788		
PEA4	0.833	14.39		
PEA5	0.667	11.719		
PEA6	0.792	13.763		

SL – Standardized loadings; CR – Composite reliability; AVE – Average variance extracted.

**Table 5.** Discriminant validity Analysis of second-order factor.

	DC	LA	PER
DC	0.787		
LA	0.592***	0.718***	
PER	0.455***	0.402***	0.755***

DC – Digital Competence, LA – Learners' Agility, PER – Perceived Learning.

### Structural model and hypotheses testing

After establishing the reliability and validity of the measurement model, the model fit indices of the structural model were tested ( Table 6). The fit indices were within acceptable range (CFI = 0.928; TLI = 0.922; RMSEA = 0.0544; SRMR = 0.0604; x2/df = 2.04).

**Table 6.** Model fit indices of the measurement and structural models.

Model	x2/df	CFI	TLI	RMSEA	SRMR
Measurement model	2.239	0.915	0.907	0.059	0.0605
Structural model	2.043	0.928	0.922	0.0544	0.0604

**Figure 2.**
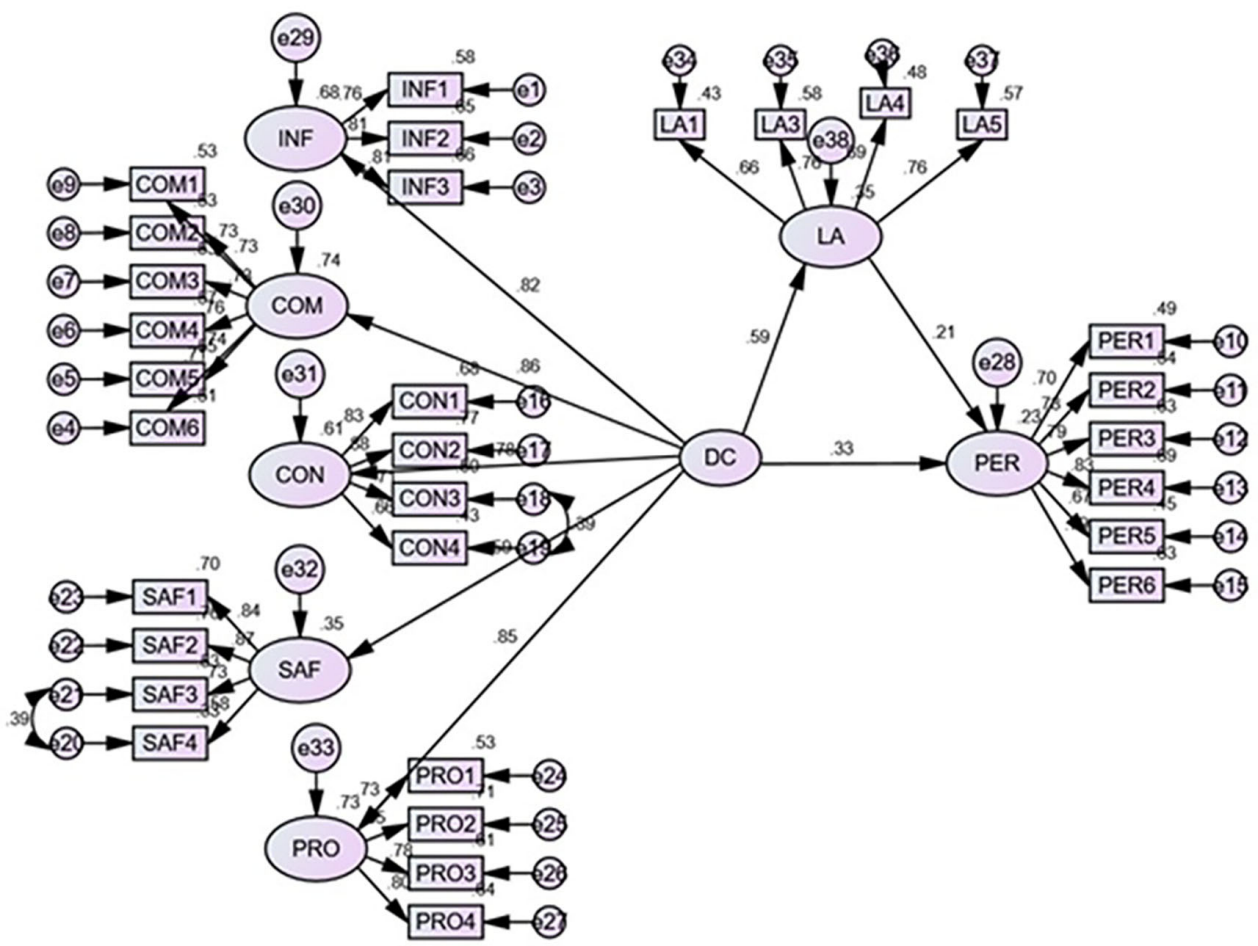
Structural model.

The structural model assessment was used to test the hypothesized relationship as conceptualized in the proposed model. This included the relationship between DC, LA, and PL. The R ^2^ values (the coefficient of determination) and beta values (path coefficients) were the parameters used to determine the strength and magnitude of the relationship between the constructs. All path relationships were statistically significant ( Figure 2).

Hypothesis 1 (H1), proposing a significant positive relationship between DC and PL, was accepted (b = 0.33; p < 0.001), indicating that higher learners’ DC leads to higher learning outcomes. Similarly, hypothesis 2 (H2), which postulated the significant positive relationship between DC and learners’ agility also found support (b = 0.59; p < 0.001), suggesting that the higher DC of learners’ leads to a higher level of learning agility. The third hypothesis (H3) that proposed the positive relationship between the learners’ agility and PL also found support (b = 0.21; p < 0.001). This significant positive path reveals that higher learners’ agility leads to higher student learning outcomes.

### Mediation analysis

The mediating effect of learning agility between DC and PL was analyzed using PROCESS macro model 4 ( Hayes, 2018). We have used the bootstrap method with 5000 re-samples to test the indirect effect as the sample size was adequate ( Zhao, *et al.*, 2010). It is found that LA has a mediating effect between their DC and PL (H4MFE-SAT-SWL: β= 0.1238, 95%, CI [0.0381, 0.216]).

## Discussion

Today higher education is becoming learner centric. The teacher assumes the role of a facilitator and catalyst to engage students in active learning with the support of innovative online teaching-learning tools and high-tech, content-rich instructional resources. Blended learning has emerged as a viable solution to manage the rapid shift to online education. In such an environment, DC plays a crucial role in students’ academic life ( Alexander *et al.*, 2016; Olszewski & Crompton, 2020). In this environment, LA (the ability to learn from the experience and adapt to new circumstances) becomes essential for integrating digital technologies into student learning and engagement in academic life.

The overarching aim of this study was to investigate the postulated association between students’ DC, LA, and PL in institutions of higher learning. To do this, we proposed four hypotheses, and the findings supported the proposed hypotheses. First, DC of students positively impacts their PL (H1). In other words, the greater the DC higher the self-perceived learning among students. E.g. the greater the self-perceived DC of students while dealing with daily digital tasks, the more likely they are to develop high self-perceived DC in areas related to their education ( Martzoukou *et al.*, 2020). However, thus far, no empirical studies in the literature have established the direct relationship between DC and PL. Second, DC significantly influences LA (H2), and the LA positively impacts students’ PL. Per the preceding statement, Kim *et al.* (2018) argued that their agility mediates the college student’s perception of DC (ability to learn and readiness to apply the acquired knowledge). The additive results revealed that LA mediated the relation between DC and PL (H4), primarily an unexplored relationship predicted in this study. In all, the findings of our study is in line with few of the past research findings ( Blau *et al.*, 2020; Heidari *et al.*, 2021; Himmelsbach *et al.*, 2019; Kim *et al.*, 2018; Mehrvarz *et al.*, 2021).

As the introduction and literature review mentioned, DC is a complex and multi-faced concept that spans several social, motivational, personal, cultural, and technical understandings. First, in the remote learning environment, students must be strongly encouraged toward self-directed learning. Researchers have a consensus that students are reflective of their learning ( Miller & Mushfiq Mobarak, 2015; Plaza-De-La-Hoz *et al.*, 2015). Their efforts in developing DC by becoming agile learners are a value addition ( Kim *et al.*, 2018). Second, in higher educational settings, educators’ technology-related knowledge, skills, and attitudes become important to improve students’ DC ( García-Vandewalle García *et al.*, 2021; Mishra & Warr, 2021). The importance of DC in students is also mirrored in the educator’s attitudes, beliefs, and professional development ( Spante *et al.*, 2018). When an educator assigns a low value to DC, students do not appreciate or acquire the soft competencies. Educators must develop a positive attitude toward imparting the digital knowledge to students ( Miller & Mushfiq Mobarak, 2015; Plaza-De-La-Hoz, *et al.*, 2015) at different levels to promote a culture of information-seeking. Third, students must be encouraged to develop self-efficacy in a safe atmosphere through the trial and error method. While researchers are investigating to develop an efficient method for improving DC among students, for a student, educators must open up for the adoption of new technologies and pedagogies. Lastly, the inclusion of course/s on DC in the higher education curriculum of all professional programs can become a ‘best practice’ of education. The dimensions of DC and their respective elements are undoubtedly applicable to a multitude of subject-specific areas ( Karsenti *et al.*, 2020), which is essentially to be adopted in present day higher education. DC can become an empowering agent to transform students into digitally literate by increasing awareness, safety behavior, digital tools, resources, and interfaces ( Alt & Raichel, 2020). As students advance through the different levels of education, DC will support students to become more autonomous in using digital technologies in academic, professional, and daily lives.

## Conclusion

Critical to the success of the transition to online education is the inevitability of having the requisite knowledge, skills, and attitudes to embrace digital technologies in a most productive manner ( Coman *et al.*, 2020; OECD, 2020). Numerous online resources facilitated students’ access, creation, and digital content sharing for collaborative education. However, every student may not possess the digital skills and competence for a seamless changeover. Though today’s learners are digitally enriched, it is evident that they are not entirely competent and agile in using the digital resources offered by the institutions. The convergence of technology, pedagogy, and an inclusive online or hybrid learning environment will push students to develop critical DC that fosters active learning and participation. Students’ prior experience with DC, where they can use a full range of digital technologies for information, communication, creation, safety, and problem-solving, will take centre stage in learning in this environment. It is documented that DC development should be initiated at an early age. Introducing a DC-based curriculum at the secondary-school level education would be ideal. However, to address the immediate needs in the post-COVID world, the integration of components of digital technologies within the higher education curriculum would support the transformation of students as “digitally literate natives”. In India, with the ‘youth bulge’ ( UNFPA India, 2021), to advocate the livelihood skill education of youth, digital enablement is vital in creating a digitally inclusive society. Towards this end, our study throws light on the necessity of developing a DC framework as a policy document that can be used in various disciplines within the landscape of higher education. This framework’s orientation should be towards using digital technology in professionally purposeful ways for lifelong learning.

## Limitations and further research

Though this study attempted to comprehend how DC and learning agility relate to and predict perceived online learning, some limitations must be noted. First, a quantitative survey is a self-report of perception of DC and learning agility. Other methods such as focus group interviews and different experimental designs can be utilized for future research. Second, a broad-based teacher DC framework must be introduced as educators have an indispensable role in implementing digital initiatives. Therefore, further studies could investigate the teaching fraternity’s DC levels and learning agility. Third, this research focused on the students in only one large private university; hence, the results may not be generalizable. Inclusion of students in diverse learning settings may be undertaken to compare the perceptions. Fourth, the demographic variables should be considered to compare the results in future investigations. Finally, this article argues the need to expand students’ understanding of the variety of DC necessary to function productively, safely and uprightly in diverse and progressively digitally mediated learning environments.

## Data availability

### Underlying data

Figshare: Students’ Digital Competence and Perceived Learning: The mediating role of Learner Agility, https://doi.org/10.6084/m9.figshare.20423496.v3 ( Mallya & Patwardhan, 2022a).

This project contains the following underlying data:

• Data.xlsx (the data set consists of four constructs: digital competence, perceived learning, learners’ agility, and self-efficacy).

### Extended data

Figshare: Digital competency_questionnaire.docx, https://doi.org/10.6084/m9.figshare.20423364.v2 ( Mallya & Patwardhan, 2022b).

This project contains the following extended data:

• Digital competency_questionnaire.docx.

Data are available under the terms of the Creative Commons Attribution 4.0 International license (CC-BY 4.0).
