# Supplementary material for: Students' Digital Competence and Perceived Learning: The mediating role of Learner Agility
Source: F1000Res. 2023 Feb 17;11:1038. Originally published 2022 Sep 12. [Version 2] doi: 10.12688/f1000research.124884.2 (PMC10840087; doi:10.12688/f1000research.124884.2)
Supplement: Peer review history

# Reviewer report: Reviewer response for version 2

**DOI:** 10.5256/f1000research.144065.r228160

**Published:** 2024-01-02

**Authors:** Mahboobe Mehrvarz

**Affiliations:** 1 Human-Computer Interaction Institute, Carnegie Mellon University, Pittsburgh, Pennsylvania, USA

Dear Authors,

 Thank you for submitting your article. I appreciate the effort and work you have put into it. Overall, your paper demonstrates a well-executed and up-to-date research study.

 In summary, your article titled "Students' Digital Competence and Perceived Learning: The Mediating Role of Learner Agility" addresses the relationship between digital competence, perceived learning, and learner agility. The study also aims to investigate the mediating role of learner agility in the relationship between digital competence and perceived learning.

 I have reviewed the manuscript thoroughly and have prepared some comments and suggestions that I believe will contribute to further improving the quality and clarity of your work.

- In the abstract, you mentioned that digital competence (DC) has a significant positive impact on perceived learning (PL) and learner agility (LA). However, it is unclear whether the mediating role of learner agility between digital competence and perceived learning was found to be significant or not.
- On the fourth page of the article, in the last line of the first paragraph, the authors mention, "Therefore, the student perspectives of cognitive, emotional, and social aspects of the learning process in a digital environment require special attention." It is unclear how this sentence is directly connected to digital competence and why it is stated.
- The introduction primarily focuses on the importance and necessity of digital competence, while your paper encompasses three variables, and the aim of your research is to examine "Students' Digital Competence and Perceived Learning: The mediating role of Learner Agility." It would be beneficial to highlight the significance and necessity of the other two variables, "Perceived Learning & Learner Agility," in relation to digital competence to provide a more comprehensive understanding of the research objectives.
- In the literature review section, it would be valuable to clearly outline the research gap addressed by your study and highlight its novel contributions to the existing literature. Additionally, providing an overview of previous studies that have examined these variables, along with their findings, would be beneficial.
- In the methodology section, including a table summarizing the direct, indirect, and total effects of the proposed relationships would enhance the clarity and visual representation of the findings.
- The second sentence of the first paragraph in result, mentions "innovative online teaching-learning tools and high-tech, content-rich instructional resources" without providing specific examples or explanations. Clarifying these descriptions by providing concrete examples of these tools and resources would enhance the reader's understanding.
- While the discussion mentions that the findings align with previous research, it would be beneficial to provide specific details or implications of these findings.

Is the work clearly and accurately presented and does it cite the current literature?

Partly

If applicable, is the statistical analysis and its interpretation appropriate?

Partly

Are all the source data underlying the results available to ensure full reproducibility?

Yes

Is the study design appropriate and is the work technically sound?

Yes

Are the conclusions drawn adequately supported by the results?

Partly

Are sufficient details of methods and analysis provided to allow replication by others?

Yes

Reviewer Expertise:

Educational Technology,Technology-Enhanced Learning, Artificial Intelligence in Education, Digital Games

I confirm that I have read this submission and believe that I have an appropriate level of expertise to confirm that it is of an acceptable scientific standard, however I have significant reservations, as outlined above.

# Reviewer report: Reviewer response for version 2

**DOI:** 10.5256/f1000research.144065.r228157

**Published:** 2023-12-20

**Authors:** Ahmad Samed Al-Adwan

**Affiliations:** 1 Electronic Business and Commerce Department, Al-Ahliyya Amman University, Amman, Jordan

Thank you for submitting the revised version of your paper. The quality of this paper has increased significantly after addressing the reviewers' comments.  However,  the hypotheses development can be strengthen and  benefit from well-established and related research. This includes but not limited to: (Al Adwan et al, 2020) ^1^

Is the work clearly and accurately presented and does it cite the current literature?

Yes

If applicable, is the statistical analysis and its interpretation appropriate?

Yes

Are all the source data underlying the results available to ensure full reproducibility?

Yes

Is the study design appropriate and is the work technically sound?

Yes

Are the conclusions drawn adequately supported by the results?

Yes

Are sufficient details of methods and analysis provided to allow replication by others?

Yes

Reviewer Expertise:

Educational technology, computer-human interaction, e-commerce

I confirm that I have read this submission and believe that I have an appropriate level of expertise to confirm that it is of an acceptable scientific standard, however I have significant reservations, as outlined above.

## References

[1] : Investigating the Impact of Social Media Use on Student’s Perception of Academic Performance in Higher Education: Evidence from Jordan dust. *Journal of Information Technology Education:Research* .2020;19: 10.28945/4661 10.28945/4661

# Reviewer report: Reviewer response for version 2

**DOI:** 10.5256/f1000research.144065.r163873

**Published:** 2023-03-15

**Authors:** Heliona Bellani (Miço)

**Affiliations:** 1 Universiteti Epoka, Tirana, Tirana County, Albania

I saw the amendments to the first version of the article, the responses of the authors regarding the suggestions, and I have no further comments to make.

Is the work clearly and accurately presented and does it cite the current literature?

Yes

If applicable, is the statistical analysis and its interpretation appropriate?

I cannot comment. A qualified statistician is required.

Are all the source data underlying the results available to ensure full reproducibility?

Yes

Is the study design appropriate and is the work technically sound?

Yes

Are the conclusions drawn adequately supported by the results?

Yes

Are sufficient details of methods and analysis provided to allow replication by others?

Partly

Reviewer Expertise:

The right to education, educational policy, teacher education, social inclusion

I confirm that I have read this submission and believe that I have an appropriate level of expertise to confirm that it is of an acceptable scientific standard.

# Response

**Published:** 2023-03-16

**Authors:** Jyothi Mallya

Thank you so much for approving the manuscript. Thank you for your time and useful feedback as well.

# Reviewer report: Reviewer response for version 1

**DOI:** 10.5256/f1000research.137121.r154642

**Published:** 2022-11-17

**Authors:** Heliona Bellani (Miço)

**Affiliations:** 1 Universiteti Epoka, Tirana, Tirana County, Albania

The authors have addressed a very current topic, digital competence in education, which has been very sensitive in recent years, especially during the COVID-19 pandemic. Considering the need for digital competence in education, the article focuses on the connection between digital competence, perceived learning, and students' learning agility.

- The authors are recommended to expand the concept of online education, which is touched on in the Introduction part.
- The authors are recommended to dwell more on the concept of perceived learning presented in the literature review, as well as on the concept of students' learning agility as a skill inside the digital area.
- In the framework of the analysis, there should be taken into consideration reasons that the students' DC is different. This can be related to the knowledge gained in pre-university education, which is addressed in the conclusions of the article but not during its analysis. Since the conclusions have affected the need for DC to be included in the curriculum of pre-university education, it is necessary to touch on how students have acquired these competencies. On the other hand, in the analysis, it is recommended to highlight the connection between digital competence and the study program followed by students, for example, the IT study program who may have higher DC than students from other fields.
- The article used the DigComp 2.1 framework to evaluate the DC profile for students and learning agility. DigComp 2.1 provides the areas of digital competence and proficiency levels. Since the article referred to the DigComp 2.1 framework, it is suggested that this should be better evidenced in the methodology used.
- It is necessary that the methodology used in the article be analyzed by psychometric experts.

The topic addressed in the article is current since society nowadays requires a set of competencies related to technology, which should start from school.

Is the work clearly and accurately presented and does it cite the current literature?

Yes

If applicable, is the statistical analysis and its interpretation appropriate?

I cannot comment. A qualified statistician is required.

Are all the source data underlying the results available to ensure full reproducibility?

Yes

Is the study design appropriate and is the work technically sound?

Yes

Are the conclusions drawn adequately supported by the results?

Yes

Are sufficient details of methods and analysis provided to allow replication by others?

Partly

Reviewer Expertise:

The right to education, educational policy, teacher education, social inclusion

I confirm that I have read this submission and believe that I have an appropriate level of expertise to confirm that it is of an acceptable scientific standard, however I have significant reservations, as outlined above.

# Response

**Published:** 2023-02-06

**Authors:** Jyothi Mallya

*The authors have addressed a very current topic, digital competence in education, which has been very sensitive in recent years, especially during the COVID-19 pandemic. Considering the need for digital competence in education, the article focuses on the connection between digital competence, perceived learning, and students' learning agility.*

- *The authors are recommended to expand the concept of online education, which is touched on in the Introduction part.*

**Our reply:**  We have added more online educational content in the introduction.**

  

- *The authors are recommended to dwell more on the concept of perceived learning presented in the literature review, as well as on the concept of students' learning agility as a skill inside the digital area.*

**Our reply: **We have added this now. 

  

- *In the framework of the analysis, there should be taken into consideration reasons that the students' DC is different. This can be related to the knowledge gained in pre-university education, which is addressed in the conclusions of the article but not during its analysis. Since the conclusions have affected the need for DC to be included in the curriculum of pre-university education, it is necessary to touch on how students have acquired these competencies. On the other hand, in the analysis, it is recommended to highlight the connection between digital competence and the study program followed by students, for example, the IT study program who may have higher DC than students from other fields.*

**Our reply:** Thank you for this suggestion; since we did not measure the knowledge gained by the students during pre-university education, we can not perform this analysis now. However, we will keep this in mind and include it in future studies. 

  

- *The article used the DigComp 2.1 framework to evaluate the DC profile for students and learning agility. DigComp 2.1 provides the areas of digital competence and proficiency levels. Since the article referred to the DigComp 2.1 framework, it is suggested that this should be better evidenced in the methodology used.*

**Our reply:** We have briefly explained the proficiency levels of DC in the methodology section.

- *It is necessary that the methodology used in the article be analyzed by psychometric experts.*

**Our reply: **We have consulted statistical experts about the methodology involved in the study. Further, we have also reported the psychometric properties of the constructs used in the study. 

*The topic addressed in the article is current since society nowadays requires a set of competencies related to technology, which should start from school.*

# Reviewer report: Reviewer response for version 1

**DOI:** 10.5256/f1000research.137121.r153846

**Published:** 2022-11-02

**Authors:** Ramachandran Sivakumar

**Affiliations:** 1 Sri Ramachandra Medical College and Research Institute (Deemed to be University), Chennai, Tamil Nadu, India

The authors have taken a very relevant topic on digital competency for research. In the current development and use of digital tools and platforms, it is critical for the user to have an understanding of a few basic elements to explore the content, use it ethically, and explore without major limitations.

- The authors could have expanded PL in the content of the article, though it is expanded in the abstract.
- The authors have developed a measuring instrument for DC using the Digicomp manual. The manual itself does not use a Likert scale to measure the attributes of DC but used a three-level rubric with an operational definition. The attributes and Likert scale have been used in the survey for students to respond. Some of the attributes like 'Engaging in citizenship through digital technologies'', 'Netiquette', 'Managing digital identity', and 'Protecting health and well-being' needs an operational definition for the respondent/student to respond appropriately.
- The authors have mentioned that oral informed consent has been obtained, but not detailed about any measures taken to orient the responders to the terminologies used in the survey and grading used. This step is likely to cause bias or median responses like moderate. However, the survey items for PL and LA were clear to read.
- As the statistical methods used were not common, a brief outline for using them in the analysis could help the reader to understand the data analysis better.
- The discussion has been to give a way forward to introduce DC in the learning environment.  

Is the work clearly and accurately presented and does it cite the current literature?

Yes

If applicable, is the statistical analysis and its interpretation appropriate?

I cannot comment. A qualified statistician is required.

Are all the source data underlying the results available to ensure full reproducibility?

Yes

Is the study design appropriate and is the work technically sound?

Yes

Are the conclusions drawn adequately supported by the results?

Yes

Are sufficient details of methods and analysis provided to allow replication by others?

Partly

Reviewer Expertise:

Physiotherapy, Education technology

I confirm that I have read this submission and believe that I have an appropriate level of expertise to confirm that it is of an acceptable scientific standard, however I have significant reservations, as outlined above.

# Response

**Published:** 2023-02-06

**Authors:** Jyothi Mallya

*The authors have taken a very relevant topic on digital competency for research. In the current development and use of digital tools and platforms, it is critical for the user to have an understanding of a few basic elements to explore the content, use it ethically, and explore without major limitations.*

- *The authors could have expanded PL in the content of the article, though it is expanded in the abstract.*

**Our reply:** Thank you for this advice; we have now added more content on the PL at the end of the introduction, as recommended by you. 

  

- *The authors have developed a measuring instrument for DC using the Digicomp manual. The manual itself does not use a Likert scale to measure the attributes of DC but used a three-level rubric with an operational definition. The attributes and Likert scale have been used in the survey for students to respond. Some of the attributes like 'Engaging in citizenship through digital technologies'', 'Netiquette', 'Managing digital identity', and 'Protecting health and well-being' needs an operational definition for the respondent/student to respond appropriately.*

**Our reply: **Since other constructs were on a 5-point Likert scale, we measured the DC on a 5-point Likert scale. The DC was measured on five levels instead of three: 1 being a low level and 5 being a high level of competence. 

  

- *The authors have mentioned that oral informed consent has been obtained, but not detailed about any measures taken to orient the responders to the terminologies used in the survey and grading used. This step is likely to cause bias or median responses like moderate. However, the survey items for PL and LA were clear to read.*

**Our reply: **Though online, the researchers circulated the questionnaire to the students in the classroom. The research team members were present to explain the constructs of the study variables.

  

- *As the statistical methods used were not common, a brief outline for using them in the analysis could help the reader to understand the data analysis better.*

**Our reply:** We have added the purpose of using these statistical analyses. 

  

- *The discussion has been to give a way forward to introduce DC in the learning environment.  *

**Our reply:** We have addressed this observation. Please refer to last paragraph of the conclusion section of the manuscript.
